# Supplementary material for: Dominant Factors Affecting Rheological Properties of Cellulose Derivatives Forming Thermotropic Cholesteric Liquid Crystals with Visible Reflection
Source: Int J Mol Sci. 2023 Feb 21;24(5):4269. doi: 10.3390/ijms24054269 (PMC10002051; doi:10.3390/ijms24054269)
Supplement: Supplementary file 1 [file ijms-24-04269-s001.zip › ijms-2023309-supplementary.pdf]

Supplementary Materials

Dominant Factors Affecting Rheological Properties of Cellulose Derivatives Forming Thermotropic Cholesteric Liquid Crystals with Visible Reflection

Yuki Ogiwara, Naoto Iwata, and Seiichi Furumi**

Department of Chemistry, Graduate School of Science, Tokyo University of Science,
1-3 Kagurazaka, Shinjuku, Tokyo 162-8601, Japan

*E-mail: n-iwata@rs.tus.ac.jp (N.I.); furumi@rs.tus.ac.jp (S.F.).

1. Supplementary Figures and Tables

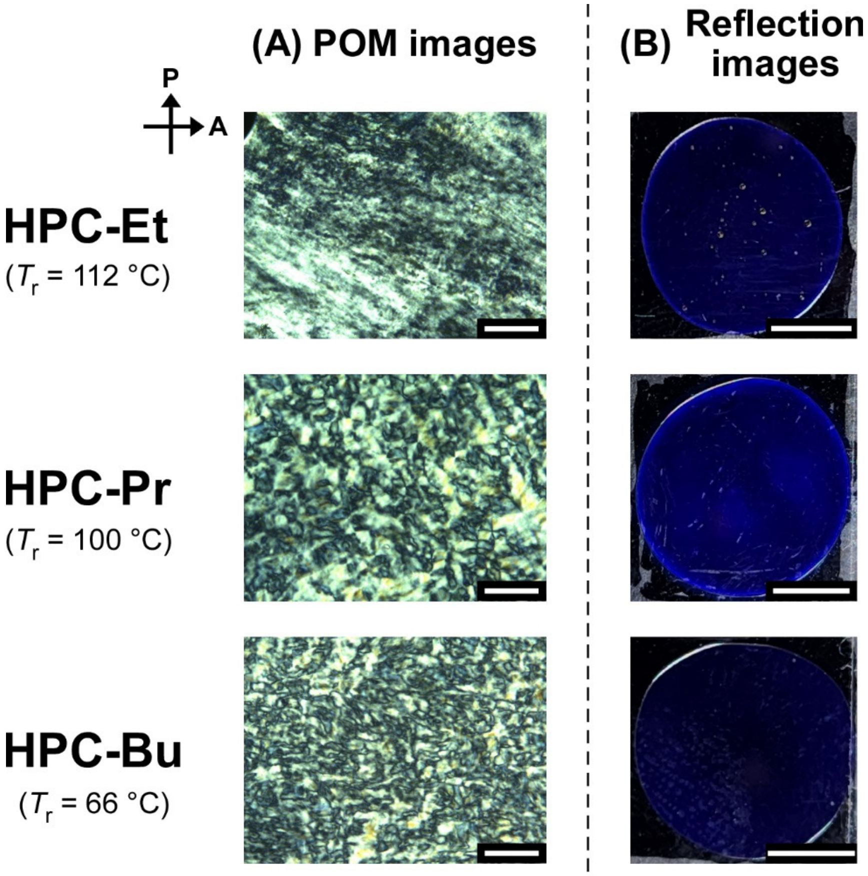

Figure S1. (A) Representative polarized optical microscopic (POM) images of HPC-Et, HPC-Pr, and HPC-Bu under crossed-Nicols. The images were taken at the reference temperatures (T_rs) for the construction of their master curves. Notice that T_rs are defined as the temperature at which each HPC derivative reflects the light at 405 nm. The white scale bars in POM images signify 100 μm. The directions of P and A refer to the axial directions of the polarizer and analyzer, respectively. (B) The reflection images of HPC-Et, HPC-Pr, and HPC-Bu taken under unpolarized white light at their T_rs. The white scale bars signify 5 mm.

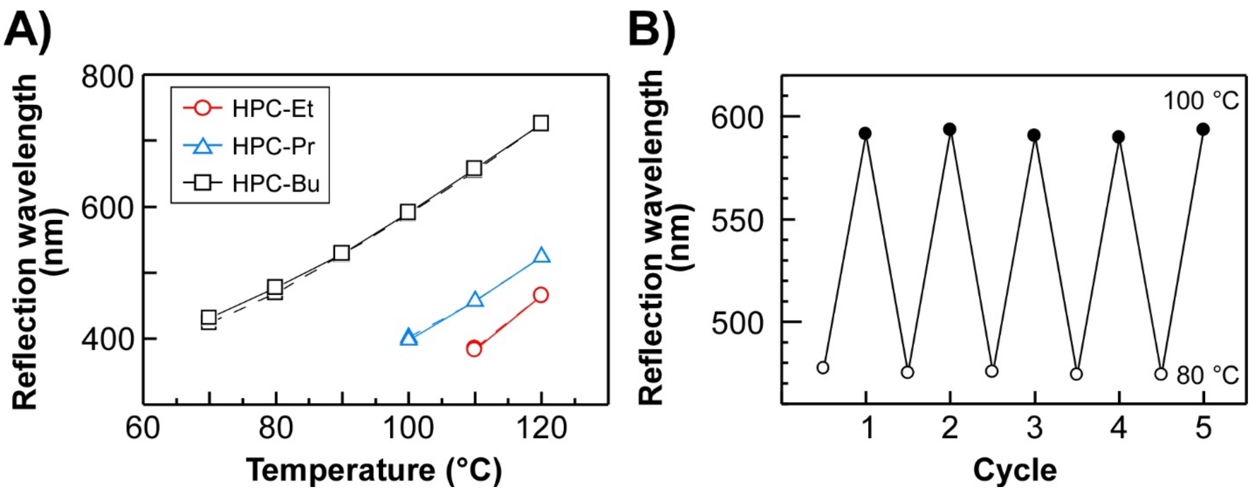

Figure S2. The reproductivity and reversibility of reflection wavelength of the HPC ester derivatives upon heating and cooling process. (A) Reflection wavelength of HPC-Et (red circles), HPC-Pr (blue triangles), and HPC-Bu (black squares) upon heating process (dotted lines) and subsequent cooling process (solid lines). Notice that the reflection wavelengths during heating are nearly identical to those during cooling, and the dotted and solid lines overlap and are difficult to distinguish. (B) Reflection peak wavelength of HPC-Bu upon repeating the cycles of temperature change between 80 °C (open circles) and 100 °C (closed circles).

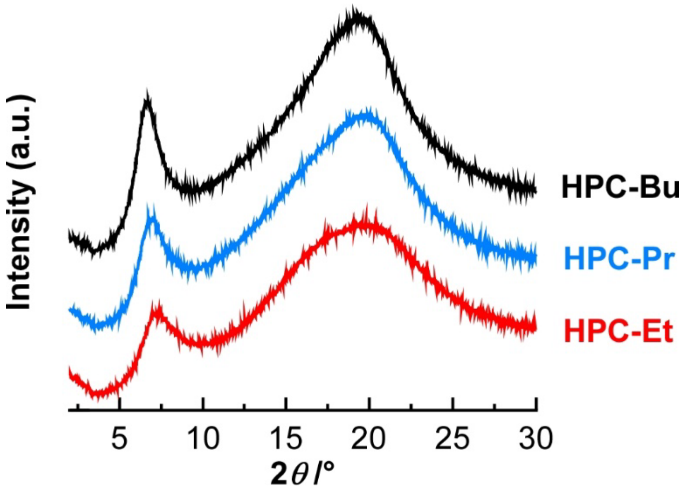

Figure S3. Wide-angle X-ray diffraction (WAXD) intensity profiles of HPC-Et (red line), HPC-Pr (blue line), and HPC-Bu (black line). We assigned the peaks appeared around $2\theta = 6°$ and halos appeared around $2\theta = 20°$ to the structure of piled layers and nematic structure in a layer, respectively. The results of WAXD measurements are summarized in Table 2.

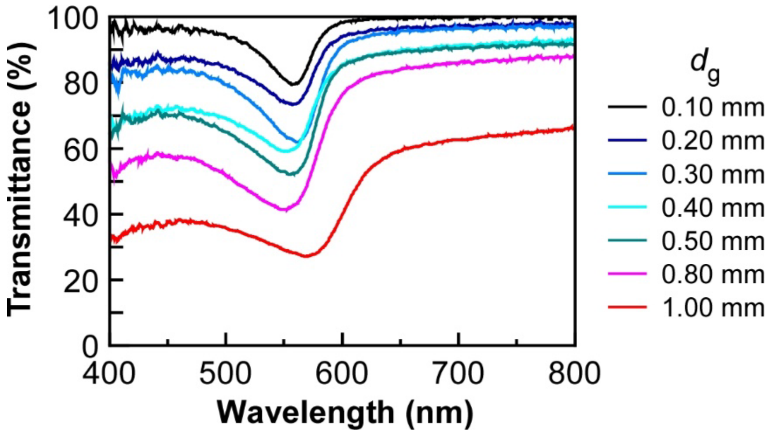

Figure S4. Changes in transmission spectrum of HPC-Pr cells at 120 °C as a function of a gap (d_g) from 0.10 to 1.00 mm. The fabrication procedure CLC cells is the same as mentioned in the Experimental Section except for the thickness of polytetrafluoroethylene film spacers. The d_g values of cells were controlled by changing the thickness of polytetrafluoroethylene film spacers. When the d_g value was thickened, the transmittance of baseline was drastically weakened and the peak width was widened presumably due to the light scattering caused by the disordered CLC orientation.

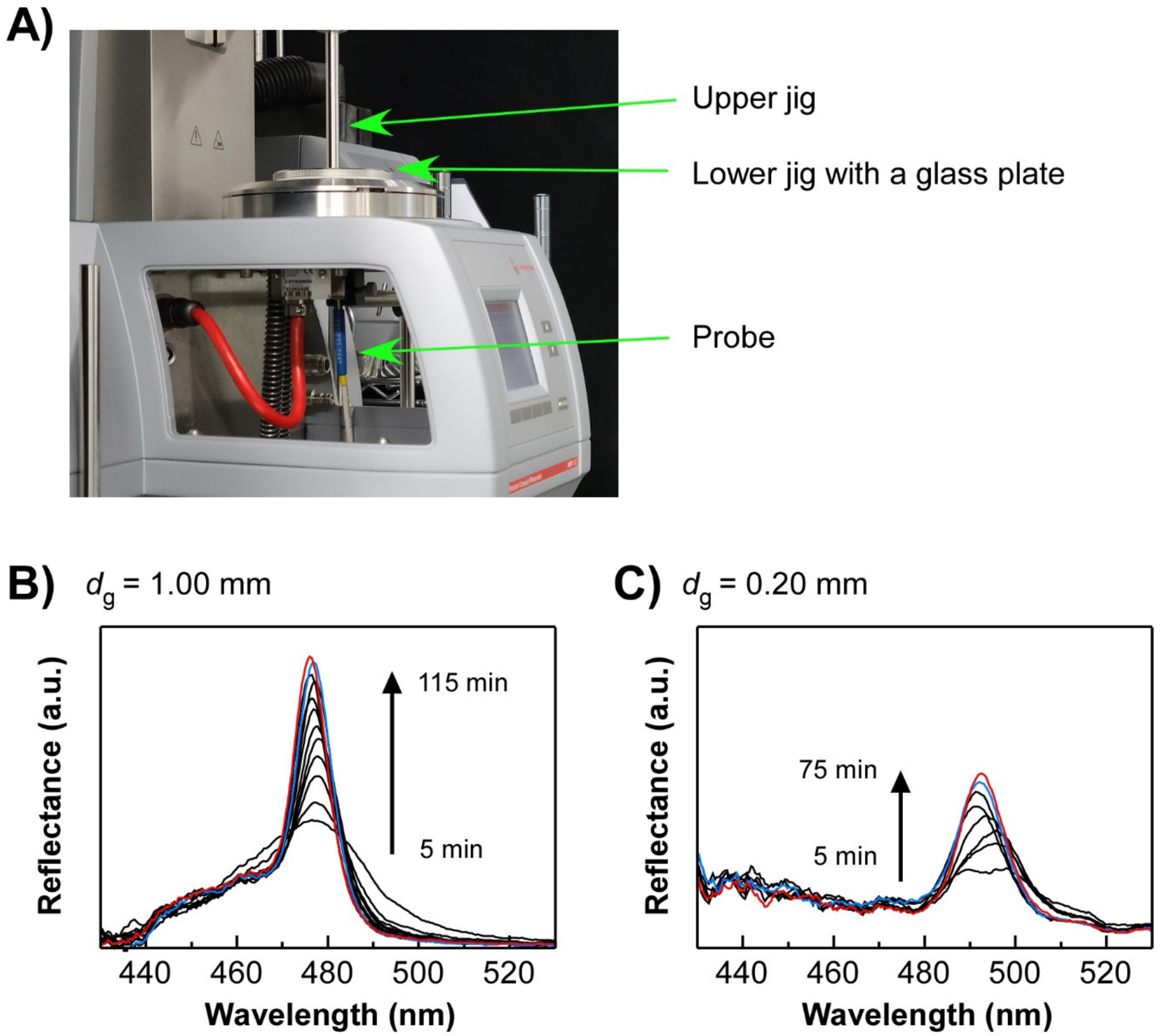

Figure S5. Reflection spectral changes of HPC-Bu after cessation of shear flow at 1.9 s^{-1} for 120 s. Recording of spectra was started in 5 min after the cessation and then every 10 min. Red and blue lines mean the result of the last measurement and the measurement 10 min before that, respectively. When there was no change in the spectra, the orientation was considered to be completed. (A) Experimental setup for rheo-optical measurement in this study. The measurements were performed using a stress-controlled rheometer (Anton Paar, MCR102) equipped with a stainless-steel parallel plate with a diameter of 25 mm as an upper jig. Though the temperature was controlled at 110 °C by the Peltier temperature control system (Anton Paar, P-PTD 200/GL), noted that it is difficult to control and keep temperature precisely in this system. The light source and spectrometer used are the same ones as mentioned in Section 2.3. in the main text. (B) Reflection spectral changes at d_g = 1.0 mm. Red and blue spectra were recorded in 115 min and 105 min, respectively. (C) Reflection spectral changes at d_g = 0.20 mm. Red and blue spectra were recorded in 75 min and 65 min, respectively.

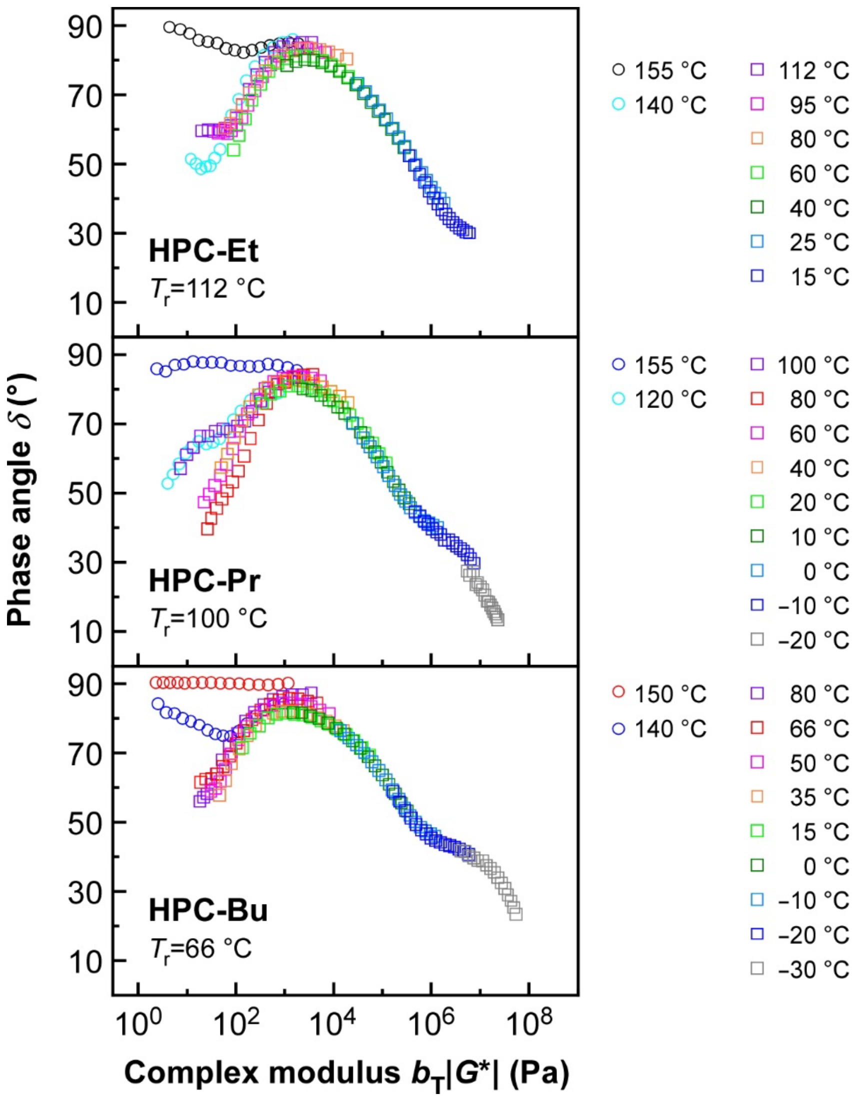

Figure S6. The van Grup-Palmen (vGP) plots for a series of HPC ester derivatives investigated in this study. The smoothness of each curve ensures the validity of time-temperature superposition principle. Open squares represent the data used for the construction of the master curves. The data plotted by open circles show the breakdown of the time-temperature superposition (TTS) principle and were not used for the construction of the master curves.

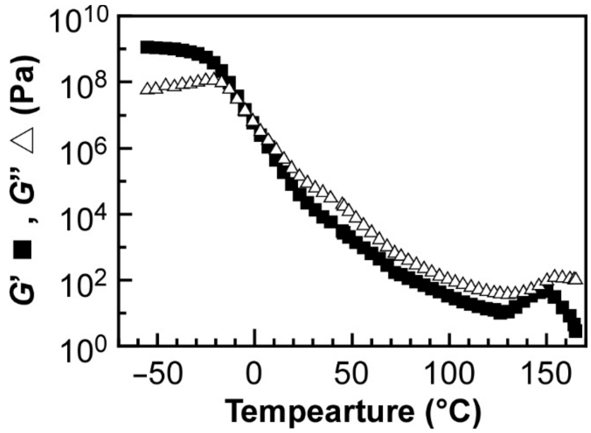

Figure S7. Temperature dependences of *G*' and *G*" at 1.0 Hz of HPC-Pr. There is a peak at 150 °C due to the phase transition to isotropic phase. No rubbery plateau was observed suggesting that HPC-Pr is not entangled.

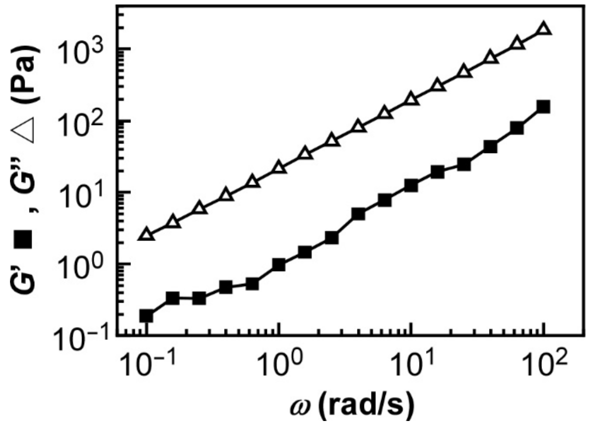

Figure S8. Frequency dependence of *G*' and *G*" of HPC-Pr at 155 °C. The slopes of *G*' and *G*" are approximately 1.0, which implies that HPC-Pr is flowing. This measurement was conducted after pre-shear treatment as with other ones.

Table S1. Characterization of HPC with various M_w and their derivatives possessing propionyl side chains (HPC-Pr-M and HPC-Pr-L). T_r values, which were the temperatures where the reflection wavelength is 405 nm, were used as reference temperature (T_r) in Figures S9A and S9B of the Supplementary Materials.

sample	*MS*	M_w (×10^4)	M_w/M_n	*DE*	T_r (°C)
HPC-M	4.00	7.81	2.24	—	—
HPC-L	4.00	13.64	2.67	—	—
HPC-Pr-M	4.00	10.48	2.00	2.99	108
HPC-Pr-L	4.00	15.40	2.42	3.01	109

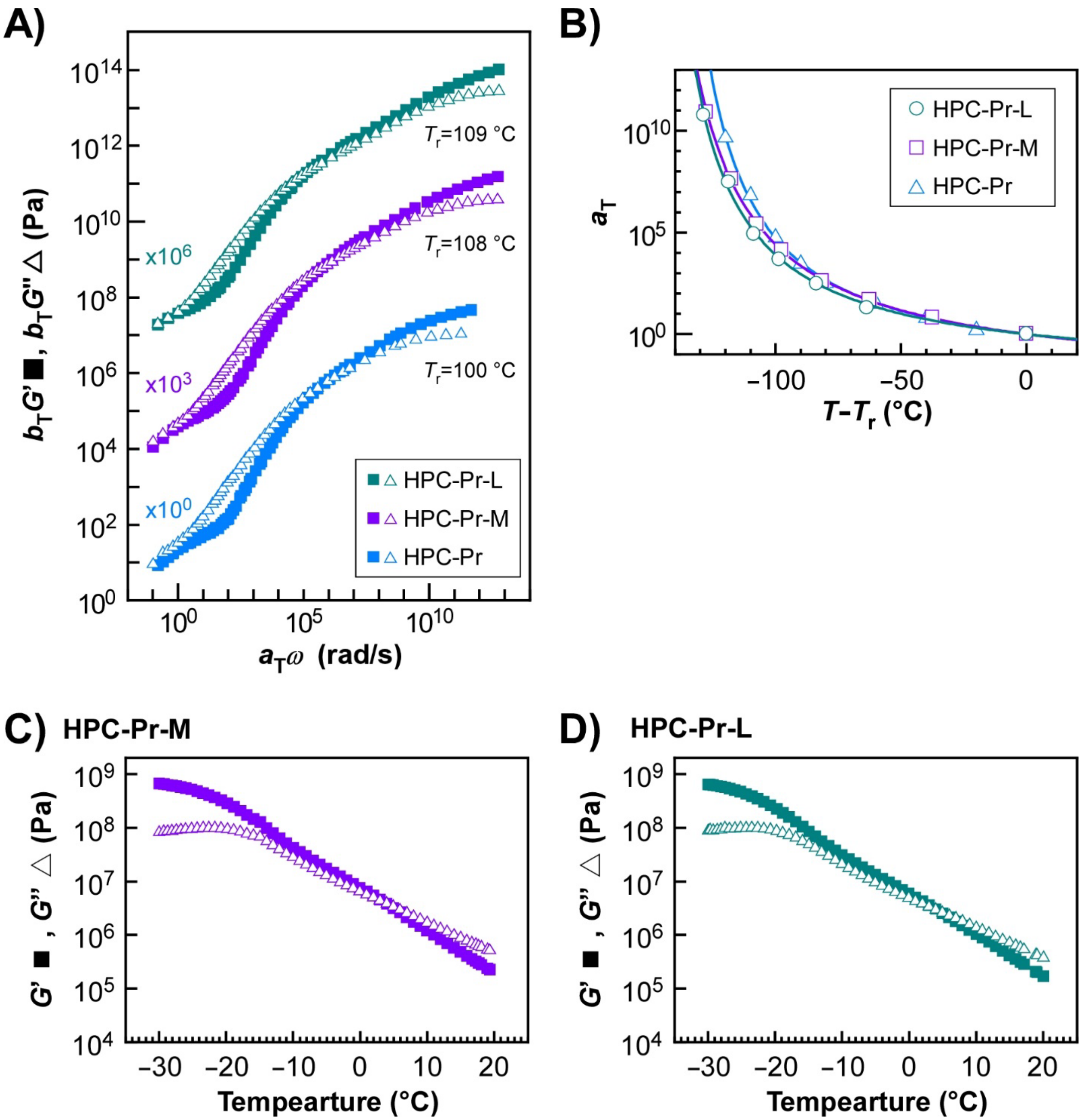

Figure S9. Rheological properties of HPC-Pr-M and HPC-Pr-L. (A) The master curves of HPC-Pr, HPC-Pr-M, and HPC-Pr-L. Their T_r values are the temperature where their reflection wavelength is 405 nm. Note that the master curves of HPC-Pr-M and HPC-Pr-L are shifted in the vertical direction of profiles by increasing both $b_T G$'and $b_T G$" values for the reader's clarity. (B) The horizontal shift factors (a_Ts) used to construct the master curves in Figure S9A. The solid lines are the curves fitted to the Williams-Landel-Ferry (WLF) equation. (C,D) Temperature dependences of G' and G" at 1.0 Hz of HPC-Pr-M (C) and HPC-Pr-L (D). Their glass transition temperatures (T_gs) were determined to be −23 °C, where the G" showed the maximum value.

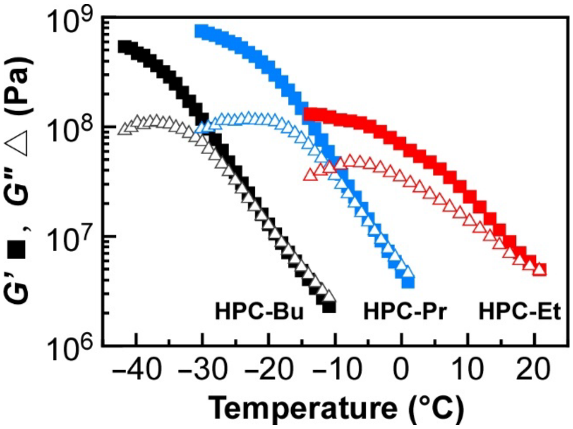

Figure S10. The temperature dependences of *G*' and *G*" of HPC-Et (red plots), HPC-Pr (blue plots), and HPC-Bu (black plots). The T_g values of HPC derivatives were determined as temperatures at which *G*" became maxima. The measurements were performed at the frequency of 1.0 Hz in the range of −50 °C to 40 °C. Only the results at the temperature range near the maximum of *G*" are presented for reader's clarity.

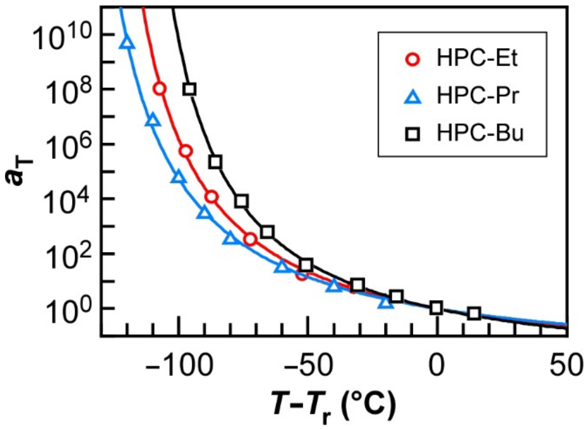

Figure S11. Changes in a_T values of HPC-Et (red circles), HPC-Pr (blue triangles), and HPC-Bu (black squares) along with WLF fit (solid lines) the T_r where all the derivatives reflect a blue light at 405 nm. The WLF parameters, c_1 and c_2, of each HPC derivative are listed in Table S2.

Table S2. The T_r values and WLF parameters of c_1 and c_2 values of HPC-Et, HPC-Pr, and HPC-Bu.

**sample**	**T_r /°C**	**c_1**	**c_2 /K**
HPC-Et	112	2.38	139
HPC-Pr	100	2.26	148
HPC-Bu	66	2.53	126

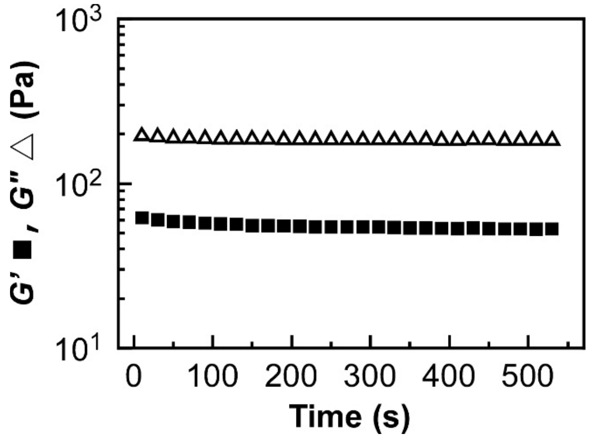

Figure S12. The time dependence of storage modulus (G') and loss modulus (G'') at 100 °C after pre-shearing HPC-Pr at a shear rate of 1 s^{-1} for 5 min. This measurement was performed at the temperature of 100 °C, at the strain amplitude of 0.7%, at the gap (d_g) of 0.50 mm, and at the frequency (ω) of 10 rad/s. This result implies that relaxation after pre-shearing is almost completed in 120 s.

2. Deviation of *DE* from ^{1}H-NMR Spectra of HPC Ester Derivatives

Here, the method to calculate *DE*, which is the average number of esterified hydroxy groups per HPC monomer unit, is explained with reference to our previous studies [S1]. If a hydroxy group in the side chain of HPC is esterified, a peak of methine proton of the hydroxypropyl group shifts to around 5.0 ppm in its ^{1}H-NMR spectra (Figure 2, right panel). Here, we defined the integrated value of the methine proton peak as A and the sum of integrated values of the peaks derived from protons of the HPC derivatives as W. The number of total protons that an HPC monomer unit has is described as

$$7 + 6MS + nDE \tag{S1}$$

where n is the number of protons in alkanoyl groups; $n = 3$ for HPC-Et, $n = 5$ for HPC-Pr, $n = 7$ for HPC-Bu, respectively. Because the ratio of the number of protons in the esterified methine group to all protons is equal to A/W, the following Equation (S2) is obtained.

$$\frac{DE}{7 + 6MS + nDE} = \frac{A}{W} \tag{S2}$$

Finally, the following Equation (S3) is derived by solving Equation (S2) for *DE*.

$$DE = \frac{A(7 + 6MS)}{W - nA} \tag{S3}$$

Supplementary Reference

[S1] Ishizaki, T.; Uenuma, S.; Furumi, S. Thermotropic Properties of Cholesteric Liquid Crystal from Hydroxypropyl Cellulose Mixed Esters. *Kobunshi Ronbunshu* **2015**, *72*, 737–745.

3. Calculation of the Activation Energy

The activation energy of the relaxation process (E_a) can be obtained from a horizontal shift factor (a_T). Here, we explained the reason why a_T show Arrhenius-type behaviors and what E_a means. The sample, which is in an excited state due to the addition of strain, returns to the ground state by the molecular motion. Usually, various mechanism of relaxation is combined, such as the motion of the center of gravity of the molecule and segmental motion of side chains. In the viscoelasticity measurements, the relaxation time (τ) of the relaxation process when molecules return to the ground state is measured. Therefore, τ means the time it takes for stress to reach e^{-1} of the initial value in the stress relaxation measurement, where e is the base of the natural logarithm. Since the inverse of τ, that is, τ^{-1}, is the rate of the relaxation process, it follows the following Arrhenius equation

$$\frac{1}{\tau} \propto \exp\left(-\frac{E_a}{RT}\right) \quad \text{(S4)}$$

where R is the gas constant, and T is the absolute temperature. Here, τ can be expressed as the product of the relaxation time at the reference temperature (τ_{ref}) and a_T.

$$\tau = \tau_{\mathrm{ref}} a_{\mathrm{T}} \quad \text{(S5)}$$

By substituting Equation (S5) into Equation (S4), Equation (S6) is obtained.

$$\frac{1}{a_{\mathrm{T}}} \propto \exp\left(-\frac{E_a}{RT}\right) \quad \text{(S6)}$$

Taking the logarithm of both sides of Equation (S6),

$$\ln a_{\mathrm{T}} = \frac{E_{\mathrm{a}}}{R} \cdot \frac{1}{T} + \mathrm{const.} \quad \text{(S7)}$$

Therefore, the value of E_a can be obtained by multiplying the slope of the regression line of the Arrhenius plot by R. In this way, E_a means the magnitude of the energy barrier for the molecules to move.
